# Black Tea Aqueous Extract Extends Yeast Longevity via Antioxidant Gene Activation: Transcriptomic Analysis of Anti‐Aging Mechanisms

**DOI:** 10.1002/fsn3.70661

**Published:** 2025-07-24

**Authors:** Jie Li, Qiyang Chen, Fuliang Xiao, Juan Yang, Sijia Zhou, Min Tang, Yujia Hou, Xiuming Zhai

**Affiliations:** ^1^ Chongqing Academy of Agricultural Sciences Chongqing China; ^2^ Engineering Research Center of Biomass Materials, Ministry of Education, School of Life Sciences and Agri‐Forestry Southwest University of Science and Technology Mianyang China; ^3^ Chongqing University Jiangjin Hospital Chongqing China

**Keywords:** anti‐aging, antioxidant capacity, black tea, chronological lifespan, *Saccharomyces cerevisiae*

## Abstract

Although tea polyphenols have antiaging potential, the molecular interplay between black tea components and cellular longevity remains unclear. This study has pioneered a dual approach combining *Saccharomyces cerevisiae* lifespan assays with comprehensive transcriptomics to dissect how black tea aqueous extract (BTAE) extends the chronological lifespan (CLS). Remarkably, BTAE induced a dose‐dependent lifespan extension (34.6% maximum at 40 μg/mL), coupled with a 62% reduction in reactive oxygen species (ROS) accumulation and enhanced superoxide dismutase/catalase/ascorbate peroxidase (SOD/CAT/APX) enzyme activities—hallmarks of antioxidant system activation. Transcriptomic profiling revealed the action mechanism of BTAE: (1) upregulation of oxidative defense arsenals (*SOD1*/*2*, *CTA1*, and *CTT1*) and mitochondrial efficiency genes (*COX10*), and (2) strategic downregulation of apoptosis triggers (*NMA111* and *AIF1*) and aging accelerators (*MTH1* and *HSPs*). Additionally, we have identified novel targets including the *OLE1*‐mediated membrane protection pathway and *IRE1*‐dependent proteostatic regulation as key longevity switches. These findings establish BTAE as a multi‐target anti‐aging modulator and offer a roadmap for translating yeast discovery into mammalian aging interventions.

## Graphical abstract

BTAE's dual‐action mechanism: Upregulation of oxidative defense arsenals (SOD1/2, CTA1, and CTT1) and downregulation of apoptosis triggers (NMA111, AIF1) and aging accelerators (MTH1, HSPs).

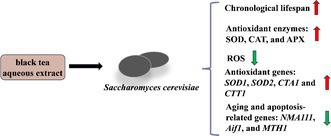

## 1 Introduction

Aging is an inevitable process characterized by a gradual decline in bodily functions once the body reaches maturity. This natural progression weakens the ability to withstand environmental stressors and increases susceptibility to age‐related diseases, such as Parkinson's disease (PD), Alzheimer's disease (AD), diabetes, cardiovascular diseases, and cancer (Moqri et al. 2023). Aging involves a complex interplay among various mechanisms operating at different levels. It is widely acknowledged that the process of aging and the onset of many diseases can be largely attributed to the harmful effects of oxidative damage caused by free radicals (Luo et al. 2020). The theory of free radicals asserts that organisms age partly because of an imbalance between the generation of reactive oxygen species (ROS) and antioxidants (Bolduc et al. 2019). In eukaryotic cells, mitochondria play a vital role in cellular bio‐oxidation and energy metabolism and serve as the primary hub for these essential processes. It should be noted that an excess of ROS can inflict harm upon mitochondria, thus resulting in cell destruction and, ultimately, contributing to the onset of aging and various diseases (Rottenberg and Hoek 2017). Organisms primarily eliminate ROS through two antioxidant systems: intrinsic enzymatic antioxidants, including catalase (CAT), superoxide dismutase (SOD), and ascorbate peroxidase (APX); and extrinsic non‐enzymatic compounds such as vitamin E and carotenoids (Harris and DeNicola 2020).

Tea ( *Camellia sinensis* L.) is recognized as the second most commonly consumed non‐alcoholic beverage worldwide, after water, and is renowned for its therapeutic properties and diverse flavors. Black tea, also known as fully oxidized tea, is renowned for its complete fermentation process. Numerous previous studies have indicated that black tea and its extracts exhibit a wide range of pharmacological effects, including anti‐inflammatory, anti‐oxidant, anti‐tumor, and anti‐cancer (Muhammad et al. 2018). The active components of black tea that contribute to its antioxidant and anti‐inflammatory properties primarily include flavonoids, phenolic acids, and methylxanthines (Li et al. 2013). Cellular senescence is a physiological response to stress triggered by sustained cellular damage resulting from chronic stressors. This phenomenon can be initiated by both cell‐intrinsic (e.g., telomere attrition) and cell‐extrinsic (e.g., nutrient stress) damages via a variety of molecular sensors (Sharma and Diwan 2022). Although preliminary studies have indicated that the bioactive constituents of tea (e.g., theaflavins and thearubigins) have an impact on biomarkers of cellular senescence, further in vivo and clinical studies are necessary to thoroughly validate these claims (Xu et al. 2021; Peng et al. 2009). Moreover, black tea extracts and their components offer protection against different age‐related ailments, such as PD (Anandhan et al. 2012), AD (Mathiyazahan et al. 2015), and diabetes mellitus (Qu et al. 2019). It has been reported that black tea extract enhances the resistance of *Caenorhabditis elegans* to osmosis, heat, and UV irradiation treatments (Xiong et al. 2014). Unfortunately, in‐depth research on the anti‐aging properties of black tea extracts remains insufficient.

*Saccharomyces cerevisiae* ( *S. cerevisiae* ) is widely used as a model organism for studying the mechanisms underlying aging, age‐related ailments, and the intricate connections linking aging with oxidative stress (Bishop and Guarente 2007). Thus far, approximately 300 proteins have been identified in *S. cerevisiae* that exhibit functions analogous to those found within the human body (Fontana et al. 2010). Additionally, the cultivation of *S. cerevisiae* is easy, and its short life span (chronological mean life span is 6–15 days) renders it an ideal model for conducting pivotal investigations into aging mechanisms and screening for anti‐aging active substances. In order to explore the effects of black tea aqueous extract (BTAE) on the aging process, we have employed *S. cerevisiae* as a model organism to comprehensively evaluate the anti‐aging properties of BTAE. Specifically, we investigated the impact of BTAE on chronological life span (CLS) and the activity of key antioxidant enzymes, including SOD, CAT, and APX. To gain deeper insight into the molecular mechanisms underlying the anti‐aging effects of BTAE, we conducted ribonucleic acid sequencing (RNA‐seq) analysis to identify the differentially expressed genes and pathways associated with aging and oxidative stress. Through this multifaceted approach, we aimed to elucidate the potential of BTAE as an antiaging agent and to uncover the molecular pathways through which it exerts its protective effects.

## 2 Materials and Methods

### 2.1 Materials and Chemicals

Black tea (Yucha 4#) was purchased from the Research Institute of Tea (Chongqing Academy of Agricultural Sciences, China). The *S. cerevisiae* BY4742 (ATCC, 201389) strain (*MATα his3*∆1 *leu2*∆0 *lys2*∆0 *ura3*∆0) was acquired from the American Type Culture Collection (Manassas, VA, USA). Sigma‐Aldrich (Shanghai, China) provided a yeast nitrogen base (YNB) without amino acids, l‐amino acids, peptone, ammonium sulfate, agar, yeast extract, or H_2_DCFDA. Yeast peptone‐dextrose (YPD) broth and other chemicals were obtained from Solebo Bio‐Tech Co. Ltd. (Beijing, China). All high‐performance liquid chromatography (HPLC) analysis standards were procured from Biopurify Phytochemicals Ltd. (Chengdu, China). The flat‐bottomed polystyrene microplates with 96 wells were acquired from Corning Inc. (Kennebunk, ME, USA).

### 2.2 Preparation of BTAE

**FIGURE 1:**
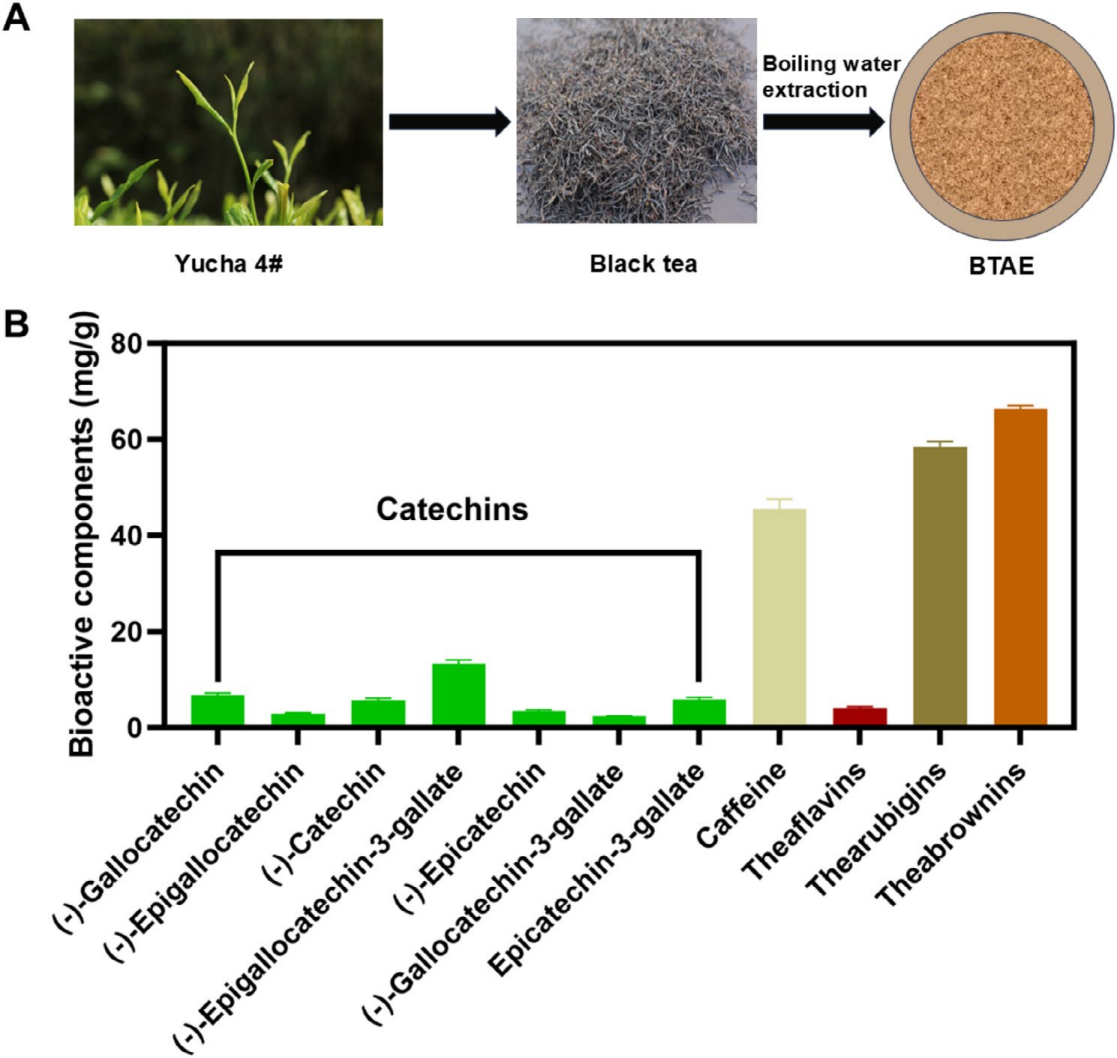
The preparation process (A) and bioactive components content (B) of the black tea extract (BTAE).

Dried black tea powder was extracted using a material‐to‐liquid ratio of 1:20 (w/v) at a temperature of 100°C for 20 min. Afterward, the mixture was centrifuged, and the residual material underwent another 10‐min extraction. The combined supernatant obtained from the two extractions was filtered. Next, the solution was subjected to evaporation by heating at 55°C using a rotary evaporator, and subsequently freeze‐dried (Figure 1A). The resulting powdered product was stored at −80°C until further use.

### 2.3 Determination of Catechins and Caffeine

Catechins and caffeine contents were determined as previously reported (Li et al. 2021). Briefly, BTAE powder (10 mg) was dissolved in 10 mL of methanol, passed through a 0.22 μm filter, and then analyzed by HPLC (Agilent 1200 series HPLC system). The constituents were separated using an Agilent C18 (250 × 4.6 mm, 5 μm) separation column under the following conditions: a column temperature of 30°C, an injection volume of 10 μL, and an analysis wavelength of 278 nm. The mobile phase, consisting of methanol (A) and water (B), was flowed at a rate of 0.8 mL/min. The gradient elution procedure was as follows: the proportion of A was decreased from 80% to 50% in the first 20 min and then increased from 50% to 75% from 20 to 25 min.

### 2.4 Determination of Theaflavins, Thearubigins, and Theabrownins

The theaflavins, thearubigins, and theabrownins were determined following the systematic analysis method described by Hua et al. (2018). Briefly, 3 g of BTAE powder was dissolved in 125 mL of distilled water, and 30 mL of tea soup was transferred to a 100 mL separating funnel. Next, 30 mL of ethyl acetate was added to the funnel and allowed to stand after shaking for 5 min. The ethyl‐acetate‐containing upper layer and aqueous (lower) layer was carefully separated, and 2 mL of the upper layer was mixed with 95% ethanol to a final volume of 25 mL, resulting in solution A. For solution B, it consisted of a lower layer (2 mL), saturated oxalic acid solution (2 mL), distilled water (6 mL), and 95% ethanol (15 mL). For solution C, the upper layer (15 mL) and 2.5% sodium bicarbonate solution (15 mL) were mixed in a 30 mL separating funnel, shaken for 30 s, allowed to stand for layering, and the upper layer was discarded. Then, 4 mL of the lower layer was removed, and 95% ethanol was added in order to reach a total volume of 25 mL. Next, 15 mL of tea soup was added to a 30 mL separating funnel, and 15 mL of n‐butanol was added. The mixture was shaken for 3 min and allowed to stand until it layered. Subsequently, the water layer (2 mL), saturated oxalic acid solution (2 mL), distilled water (6 mL), and 95% ethanol (15 mL) were mixed to obtain solution D. The absorbance of solutions A, B, C, and D was measured at 380 nm and recorded as *E*_A_, *E*_B_, *E*_C_, and *E*_D_, respectively. The results were calculated using equations (Moqri et al. 2023; Luo et al. 2020; Bolduc et al. 2019).

(1) $$\text{Theaflavins}=2.25\times E_{C}\times 100\%/m$$

(2) $$\text{Thearubigins}=7.06\times \left(2E_{A}+2E_{B}-E_{C}-2E_{D}\right)\times 100\%/m$$

(3) $$\text{Theabrownin}=2\times E_{D}\times 7.06\times 100\%/m$$

### 2.5 Cell Activation and Viability Assay

The yeast cells were activated on YPD agar plates containing 1% peptone, 0.5% yeast extract, 1.4% agar, and 2% dextrose at 30°C for 2 days. A single colony was transferred to YPD liquid medium (1.0 mL) in a sterile 10 mL centrifuge tube, which contained 1% yeast extract, 2% peptone, and 2% dextrose, and it was cultured at 30°C and 200 rpm for 2 days. The YPD liquid medium was diluted with sterilized Milli‐Q grade water (18 mΩ) at a ratio of 1:10, and then refrigerated at 4°C for 2 days.

The diluted culture (5 μL, ≈1 × 10^4^ cells) and extract (2 μL) were added to 993 μL of synthetic‐defined (SD) media, and then it was kept at 30°C and 200 rpm (Guo et al. 2019). Extracts of different final concentrations (0.5–160 μg/mL) were co‐cultured with yeast, and the cell count was measured by the plate counting method after 24 h of cultivation. The test extract concentrations that exhibited no alterations in cell viability compared to the control group (containing DMSO at a final concentration of 0.2%) were chosen for subsequent investigation.

### 2.6 Determination of Chronological Lifespan (CLS)

The CLS determination of yeast was conducted following the procedure outlined by Wu et al. (2011), with slight adjustments. Briefly, SD medium containing different concentrations of BTAE was incubated at 30°C without changing the medium during the experiment. After 2 days of incubation in the maturing medium, the cells entered the stationary phase, marking the initiation of the aging process. Samples were collected at different age points (2, 5, 8, 11, 14, and 17 days), and 5 μL of the sample and 95 μL of YPD medium were transferred into a 96‐well flat‐bottom microplate. The cellular population was determined using a microplate reader (Varioskan Flash; Thermo Scientific, Waltham, MA, USA), and the optical density at OD_660_ was measured at 10‐min intervals for 24 h. The relative OD, relative survival (Vn), and area under curve (AUC) were calculated according to Equations ((4), (5), (6), (7), (8), (9)):

(4) $$D_{t}=\frac{\ln\left(2\right)}{\frac{\ln\left({\mathrm{OD}}_{2}\right)-\ln\left({\mathrm{OD}}_{1}\right)}{t_{1}t_{2}}}$$

(5) $$\lambda =\frac{\frac{\ln\left(2\right)}{\bar{{\mathrm{Dt}}_{n}}}\times \frac{{\mathrm{OD}}_{<0.3}}{{\mathrm{OD}}_{>0.3}}\times t_{\mathrm{OD}<0.3}-\frac{\ln\left(2\right)}{\bar{{\mathrm{Dt}}_{n}}}\times t_{\mathrm{OD}>0.3}}{1-\frac{{\mathrm{OD}}_{<0.3}}{{\mathrm{OD}}_{>0.3}}}$$

(6) $$\begin{array}{c} \mathrm{\Delta t}=\left(\frac{t_{\mathrm{OD}>0.3}-t_{\mathrm{OD}<0.3}}{{\mathrm{OD}}_{>0.3}-{\mathrm{OD}}_{<0.3}}\times 0.3+t_{\mathrm{OD}>0.3}-\frac{t_{\mathrm{OD}>0.3}-t_{\mathrm{OD}<0.3}}{{\mathrm{OD}}_{>0.3}-{\mathrm{OD}}_{<0.3}}\times {\mathrm{OD}}_{>0.3}\right)\\ -t_{\mathrm{OD}=0.3,2\mathrm{day}} \end{array}$$

(7) $$V_{n}=\frac{1}{2^{\frac{{\mathrm{\Delta t}}_{n}}{\lambda }}}\times 100$$

(8) $$\text{Relative}\;\mathrm{OD}=\frac{V_{n}}{V_{0}}\times 100$$

(9) $$\mathrm{AUC}=\sum_{2}^{n}\left(\frac{V_{n-1}+V_{n}}{2}\right)\left({\mathrm{day}}_{n}-{\mathrm{day}}_{n-1}\right)$$

Where OD_1_ and OD_2_ are continuous OD measurements, and *t*_1_ and *t*_2_ are the time intervals between the measurements. *t*_OD = 0.3_ refers to the time at which the OD value in the growth curve reached 0.3. *t*_OD>0.3_ and *t*_OD<0.3_ respectively indicate adjacent time points when the OD value is below 0.3 and above 0.3, respectively. The average doubling times ($\bar{{\mathrm{Dt}}_{n}}$) are defined as doubling times (Dt) only within the OD value range of 0.2–0.5. For relative survival, the initial age (day 2) was defined as 100% viable.

### 2.7 Determination of Intracellular ROS Scavenging

Intracellular ROS levels in the yeast cells were quantified using the method described by Guo et al. (2019). To ensure extract‐free conditions during ROS measurement, Day‐2 aging cultures were centrifuged (5000 rpm, 5 min), washed three times with PBS containing protease inhibitors, and resuspended in fresh Tris/HCl buffer (50 mM, pH 7.5). Cells were then incubated with 2 μL H_2_DCFDA (5 mM) for 1 h at 30°C, followed by two additional washes to remove residual probe. Subsequently, cells were lysed with chloroform (20 μL) and SDS (0.1%, 10 μL), agitated (200 rpm, 30 min) to release fluorescent adducts, and pelleted by centrifugation (5000 rpm, 5 min). Fluorescence intensity was determined at 480/520 nm (excitation/emission) using a microplate reader.

### 2.8 Determination of Antioxidant Enzyme Activity

The yeast cells were cultured and harvested 2 days post‐treatment, with or without black tea extracts (*n* = 3). Cells were ground in liquid nitrogen, weighed, and extracted using SOD, CAT, POD, and APX assay kits (Sino Best Biological Technology Co. Ltd., Shanghai, China). Enzyme activity was measured according to the manufacturer's instructions, and protein concentrations were assessed using a commercially available BCA assay kit (Auragene, China).

### 2.9 RNA‐Seq

Total RNA isolation was conducted with TRIZOL reagent (Invitrogen), followed by dual‐quality verification through spectrophotometric analysis (NanoPhotometer, IMPLEN) and electrophoretic assessment (Bioanalyzer 2100, Agilent). mRNA libraries were constructed with NEBnext UltraTM kits (Illumina‐compatible) and sequenced on a Novaseq platform (150 bp paired‐end). After evaluating raw data quality (Q20/Q30, GC content), clean reads were aligned to the reference genome (Hisat2 v2.0.5) and quantified (FeatureCounts v1.5.0‐p3), with FPKM normalization incorporating transcript length and sequencing depth. Differential expression analysis employed DESeq2 (v1.16.1), applying negative binomial models and Benjamini‐Hochberg correction (FDR‐adjusted *p* < 0.05). Significant genes underwent functional profiling via clusterProfiler, including GO term and KEGG pathway enrichment to elucidate biological relevance in the study context.

### 2.10 qRT–PCR

In order to confirm the transcriptomic results, we conducted SYBR Green‐based quantitative real‐time PCR (qPCR) assays in triplicate using a StepOne Plus system (Applied Biosystems). Six differentially expressed genes (DEGs) were selected for validation, with their respective primers provided in Table S1. Gene expression fold changes were determined via the 2^−ΔΔCt^ method (Schefe et al. 2006) relative to actin as the internal control.

### 2.11 Statistical Analysis

Results are expressed as mean ± standard deviation (SD, *n* = 3). Statistical analyses were performed with IBM SPSS Statistics 25, employing either one‐way ANOVA with Duncan's post hoc test or independent samples t‐test for comparisons. Differences were deemed significant at *p* < 0.05.

## 3 Results and Discussion

### 3.1 Analysis of BTAE

Various forms of tea extracts with good antioxidant activities have been increasingly explored as anti‐aging agents (Luo et al. 2023). Tea‐based products are common in the food, pharmaceutical, and cosmetic fields, and some have been commercialized (Wei et al. 2022). Polyphenolic compounds in tea, which are important secondary metabolites with antioxidant functions, may delay aging. Multiple studies have shown that phenolic compounds in tea extracts are the main active ingredients with anti‐aging activity (Wei et al. 2022). Black tea contains various polyphenolic compounds, including theabrownins, thearubigins, theaflavins, catechin polymeric pigments, and catechins (Li et al. 2013). Caffeine is a highly stable alkaloid that remains unaltered during fermentation. In the present study, the catechin and caffeine contents of BTAE were identified by comparing the chromatographic retention time with that of reference standards using HPLC. As shown in Figure 1B, seven catechins were identified in the BTAE, ranging from 2.29 to 13.31 mg/g. Among these catechins, (−)‐epigallocatechin‐ 3‐gallate (13.31 mg/g), (−)‐gallocatechin (6.69 mg/g), and (−)‐epicatechin‐3‐gallate (5.77 mg/g) were the major catechins in BTAE. Theaflavins, thearubigins, and theabrownin are water‐soluble compounds derived from the polymerization of tea polyphenols (Zhao et al. 2020). In this study, theabrownin in BTAE was the highest, at 66.34 mg/g, followed by thearubigins (58.45 mg/g) and theaflavins (4.1 mg/g). Previous studies have found relatively high phenolic compound content in black tea extracts (e.g., 220.02 mg GAE/g extract) (Tandale et al. 2022). Catechins, theaflavins, and thearubigins in black tea are responsible for various biological activities such as antioxidant, anti‐microbial, anti‐inflammatory, and anti‐atherosclerotic activities (Luo et al. 2023; Zhang et al. 2019). In addition, research on tea extracts (containing phenolic compounds) and their antioxidant and anti‐aging activities has shown their potential in regard to delay aging (Wang et al. 2024). However, the effects of tea on senescent aging remain unclear. Thus, *S. cerevisiae* was used as an aging research model to investigate the anti‐aging activity of BTAE (Fontana et al. 2010).

### 3.2 BTAE Increased the CLS of *S. cerevisiae*

**FIGURE 2:**
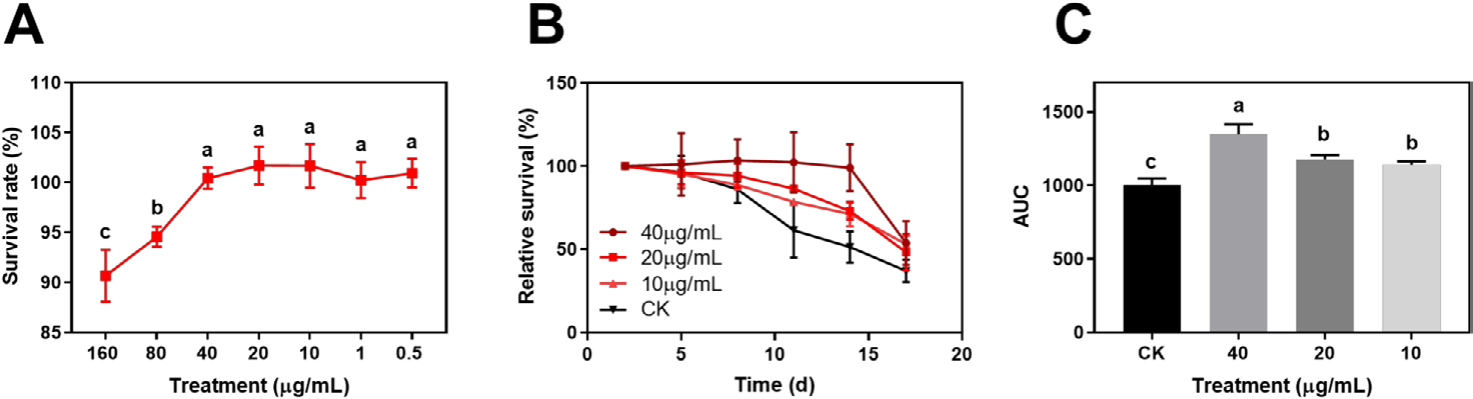
Effect of the BTAE on cell viability, survival curves, and area under the survival curve (AUC) of yeast (BY4742). Results were expressed as means ± SD, *n* = 3 experiments. Different letters represent significant differences at *p* < 0.05.

The cytotoxic effects of BTAE on *S. cerevisiae* after 48 h of exposure were assessed using the plate counting method (Figure 2A). The results indicated that BTAE concentrations ranging from 0.5 to 40 μg/mL had no detrimental effects on cell viability, and therefore these concentrations were employed in subsequent studies. At concentrations ranging from 0.1 to 10 μg/mL, the extract of Assam tea demonstrated negative cytotoxicity (Kanlayavattanakul et al. 2024). Chronological Longevity (CLS), the time non‐dividing yeast cells stay alive in the quiescent phase, is often used to monitor metazoan lifespan (Fabrizio and Longo 2008). To assess the impact of BTAE on the chronological lifespan of *S. cerevisiae* , the BY4742 strain was treated with 40, 20, 10, and 0 μg·mL^−1^ BTAE at the stationary phase for 17 days, and cell viability was monitored over time. As shown in Figure 2B,C, BTAE significantly increased the CLS. In comparison to the control group, the lifespan of the 10, 20, and 40 μg·mL^−1^ of BTAE treatment groups was extended by 13.9%, 17.7%, and 34.6%, respectively, which was consistent with previously reported results that the use of pigment rice bran ethanol extract significantly extended yeast lifespan (Sunthonkun et al. 2019). Yeast cells may die via accidental cell death (ACD) or regulated cell death (RCD). Harsh environmental factors (e.g., temperature and radiation) lead to ACD, whereas physiological conditions such as metabolic waste accumulation during yeast cell growth result in RCD. ACD disintegrates the cell structure and ruptures the plasma membrane, causing necrosis, while RCD is an apoptotic programmed death in microorganisms (Carmona‐Gutierrez et al. 2018). Polyphenols have been proven to defend against free radicals and radiation, as well as assist endogenous antioxidant systems in scavenging them (Pandey and Rizvi 2009). When compared to the untreated yeast, BTAE treatment extended the lifespan of brewing yeast, which might be related to the abundant antioxidant properties of polyphenols in BTAE, which support cellular longevity pathways and reduce the oxidative stress markers associated with aging (Luo et al. 2021).

### 3.3 Transcriptomic Sequence and Key Pathways Analysis

To investigate the molecular mechanism by which BTAE prolonged the CLS of yeast, transcriptomics analysis was performed on yeast samples treated with 20 μg/mL BTAE (BTAE group) or untreated (CK group) after 2, 8, and 17 days. This study yielded 756.41 M raw reads from 18 sequencing libraries (three replicates per group), with 738.04 M high‐quality clean reads obtained following quality control and splice removal (Table S1). Additionally, Q20 for all 18 sequencing libraries was above 97%, and Q30 exceeded 91% (Table S2), suggesting that the high quality of the sequencing results was suitable for subsequent reference genome alignment and bioinformatics analysis.

**FIGURE 3:**
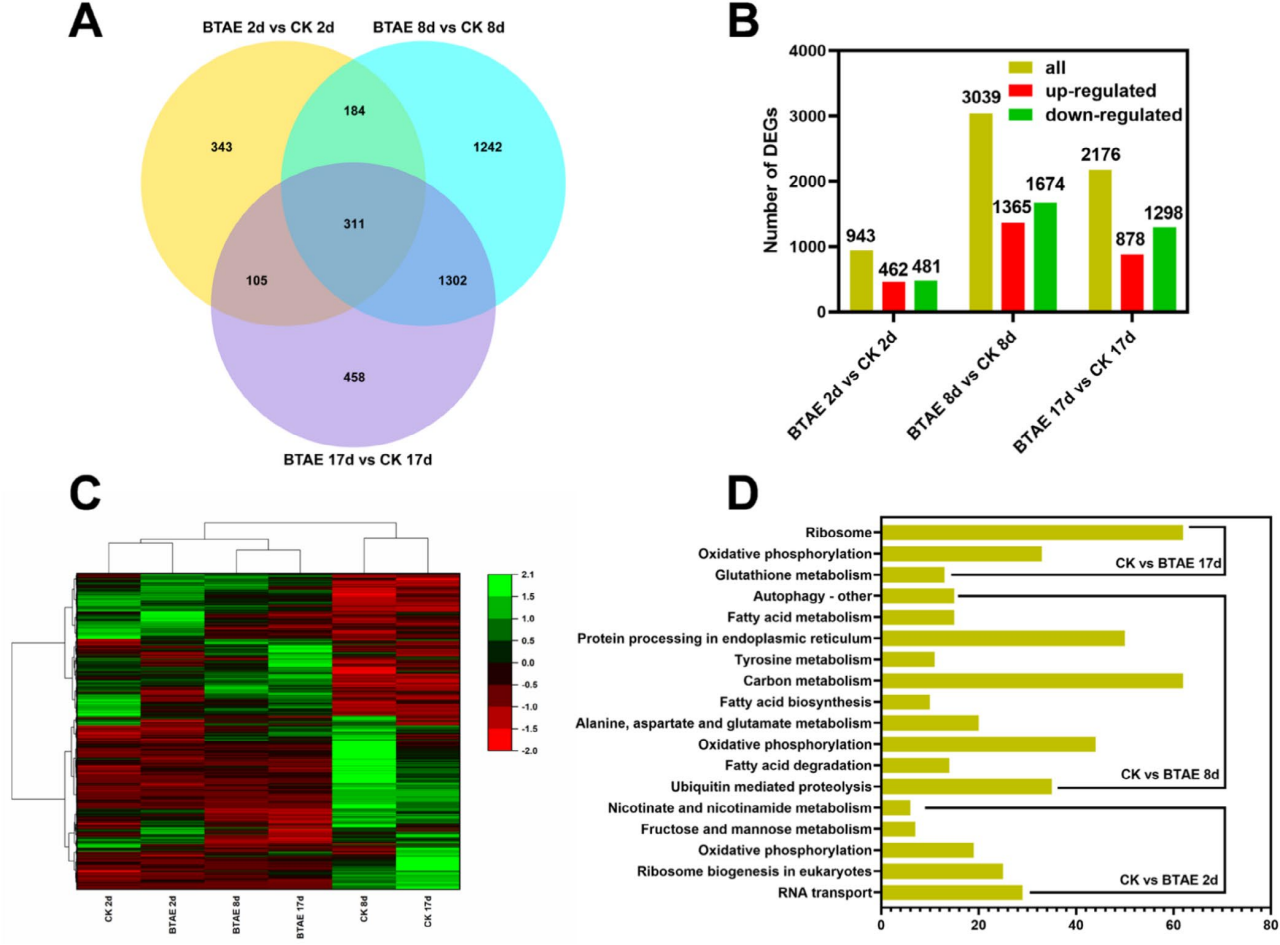
Overview of the RNA‐Seq analysis of yeast. (A) Venn diagram. (B) Number of DEGs in BTAE 2d vs. CK 2d, BTAE 8d vs. CK 8d, BTAE 17d vs. CK 17d. (C) Clustering heat map of DEGs at 2, 8, and 17 days. (D) KEGG pathway classification of the DEGs.

As shown in Figure 3A,B, 943 (462 up‐regulated and 481 down‐regulated), 3039 (1365 up‐regulated and 1674 down‐regulated), and 2176 (878 up‐regulated and 1298 down‐regulated) DEGs were identified between the BTAE 2d vs. CK 2d group, BTAE 8d and CK 8d group, and BTAE 17d and CK 17d group, respectively. The performed clustering analysis indicated that DEG abundance changed significantly with growth time and that BTAE treatment had a pronounced effect on their expression (Figure 3C). GO and KEGG enrichment analyses were performed on the DEGs. These DEGs were significantly enriched in 88 GO terms, with 61 in biological processes, 20 in molecular functions, and seven in cellular components (Table S3). Moreover, several GO terms related to aging, such as the oxidation–reduction process (GO:0055114), generation of precursor metabolites and energy (GO:0006091), pyruvate metabolic process (GO:0006090), and oxidoreduction coenzyme metabolic process (GO:0006733), were observed. KEGG analysis revealed that the DEGs were primarily enriched in RNA transport and ribosome biogenesis in eukaryotes (Table S4 and Figure 3D).

**FIGURE 4:**
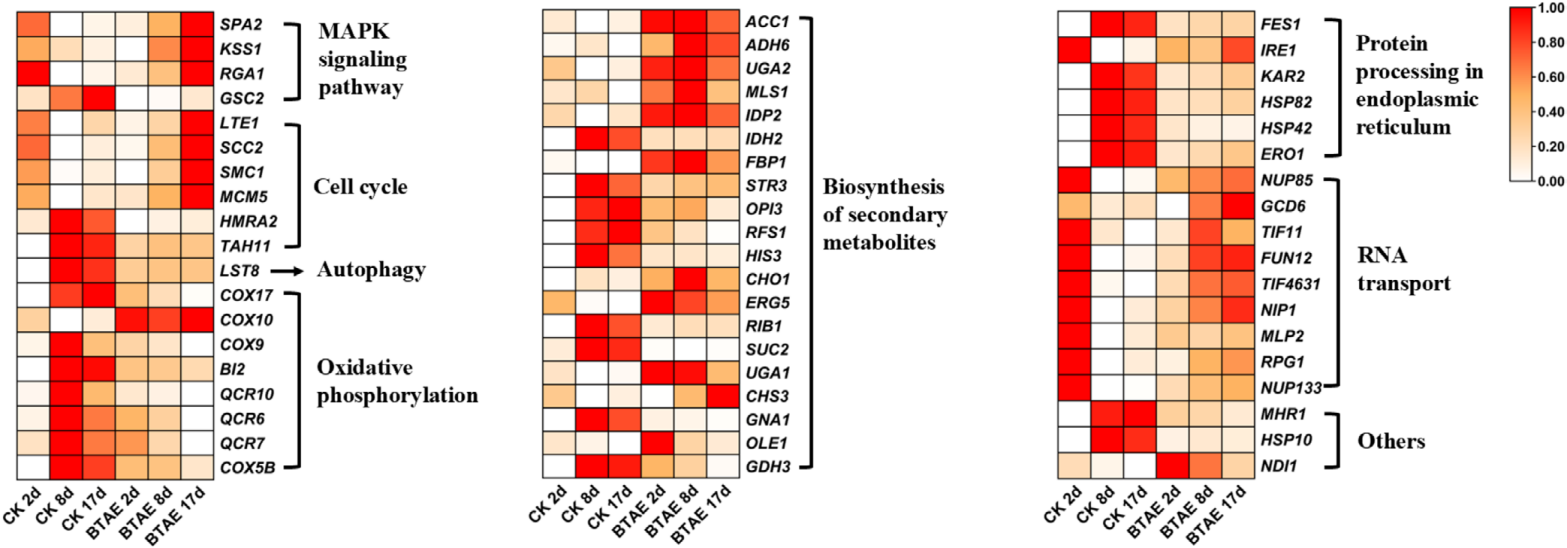
Part of common genes in three comparisons.

As shown in Figure 3A, 311 genes were common among the three comparisons, and the expression levels of the key functional genes were further identified (Table S5 and Figure 4). Polyphenols can exert anti‐oxidant, anti‐inflammatory, and apoptotic effects, and modulate epigenetic changes, thereby delaying the aging process. The expression levels of DEGs in several key pathways, including the mitogen‐activated protein kinase (MAPK) signaling pathway, cell cycle, autophagy, and biosynthesis of secondary metabolites, are presented in Figure 4. For “cell cycle” pathway, *low temperature essential 1* (*LTE1*), *sister chromatid cohesion 2* (*SCC2*), *stability of mini‐chromosomes 1* (*SMC1*), and *mini‐chromosome maintenance 5* (*MCM5*) were up‐regulated, and *homothallism right A2* (*HMRA2*) and *topo‐A hypersensitive 11* (*TAH11*) were down‐regulated under BTAE treatment at 17 days. *LTE1* is a mitotic regulator that has long been proposed to function as a guanosine nucleotide exchange factor (GEF) for the termination of M phase 1 (Tem1), a small guanosine triphosphatase that regulates the activity of the *Saccharomyces cerevisiae* mitotic exit network. An increase in *HMRA2* gene silencing led to an extension of the short‐term lifespan of the double mutant strain (Cardona et al. 2011). In the “Oxidative phosphorylation” pathway, eight DEGs were annotated. Of these, one gene was found to be up‐regulated and seven genes were down‐regulated following BTAE treatment. For “biosynthesis of secondary metabolites” pathway, eleven genes such as *isocitrate dehydrogenase NADP‐specific 2* (*IDP2*), *oleic acid requiring 1* (*OLE1*), *utilization of gamma‐aminobutyrate 1* (*UGA1*), and *UGA2* were up‐regulated by BTAE at different time in yeast cells. *Transcriptional activator of oleate genes 1* (*Tog1*) regulates *IDP2* gene expression to regenerate the antioxidant cofactor nicotinamide adenine dinucleotide phosphate (NADPH) for oxidative stress resistance (Soontorngun 2016). *OLE1* encodes yeast‐9 fatty acid desaturase, which catalyzes the production of monounsaturated fatty acids, such as palmitoleic (16:1) and oleic (18:1) fatty acids. Importantly, the antioxidant effect of OLE1 is independent of antioxidant enzymes and glutathione 1 (GSH1), but rather reduces oxidative stress by inhibiting lipid peroxidation and protecting the yeast cell membrane from damage (Huang et al. 2018). While Kamei et al. (2011) in their study observed lifespan extension via *UGA1* deletion in a replicative lifespan (RLS) model, this finding aligns with our CLS observations, suggesting a potentially conserved role for *UGA1* in yeast longevity regulation, despite the mechanistic differences between RLS and CLS. However, the expression levels of nine DEGs–*glutamate dehydrogenase 3* (*GDH3*), *isocitrate dehydrogenase 2* (*IDH2*), *sulfur transfer 3* (*STR3*), and *sucrose 2* (*SUC2*)–were inhibited by BTAE at different times. *Saccharomyces cerevisiae* cells lacking *GDH3* show sensitivity to thermal and oxidative stress as well as oxidative stress‐dependent accumulation of ROS, which leads to apoptotic cell death (Mara et al. 2018). In “protein processing in endoplasmic reticulum” pathway, the expression of *inositol requiring 1* (*IRE1*) was up‐regulated by BTAE at 8 and 17 days, while the expression levels *of factor exchange for ssa1p* (*FES1*), *karyogamy 2* (*KAR2*), *heat shock protein 82* (*HSP82*), *HSP42*, and *endoplasmic reticulum oxidation 1* (*ERO1*) were inhibited. Chadwick et al. (2019) reported that the progressive deterioration of proteostasis and the accumulation of toxic misfolded proteins are closely linked to cellular aging. Deletion of IRE1 shortens the chronological lifespan. The *Hsp82* ^*S485A*^ strain has impaired membrane‐related protein transport, and its cell size does not increase during quiescence when compared to the logarithmic phase, and it cannot survive under starvation conditions. Oxidation inactivated the activity of Fes1 activity, which alters Hsp70 activity, thereby maintaining protein‐folding homeostasis in a suboptimal cellular folding environment (Nicklow and Sevier 2020). Oxidative phosphorylation, which involves four enzyme complexes and ATP synthase, generates the primary cellular ATP pool and plays a critical role in sustaining physiological tissue and cellular growth (Chu et al. 2021). Cytochrome coxidase (COX) is a key regulator of this process. In cell‐based experiments, the silencing of *COX5B* repressed cell growth. *Cox10* is essential for heme A biosynthesis, and its deficiency leads to hematopoietic diseases (Li et al. 2022). In this study, BTAE downregulated the expression of *COX9*, *COX17*, *COX5B*, *ubiquinol‐cytochrome c oxidoreductase* (*QCR6*), *QCR7*, and *QCR10*, while simultaneously upregulating the expression of *COX10*. Defective QCR7 results in diminished recruitment of inflammatory cells and a reduction in the virulence of *Candida albicans* infections in vivo (Zeng et al. 2023). Abundant evidence has proven that phytochemicals extend the lifespan by reducing oxidative stress, suppressing low‐grade chronic inflammation and inducing autophagy. In addition, crosstalk and interactions between these events were observed at both the cellular and molecular levels. Oxidative damage induced by free radicals is regarded as a primary contributor to aging and many diseases (Zhou et al. 2018). In the aging yeast, autophagy is highly dependent on acetyl‐CoA carboxylase (ACC1) activity. However, the expression levels of *lethal with sec thirteen* (*LST8*) involved in autophagy was found to be down‐regulated. LST8 acts together with both target of rapamycin 1 (TOR1) and TOR2 to negatively regulate the synthesis of glutamate and glutamine The dysregulation of inflammatory homeostasis frequently occurs alongside the aging process, with inflammation itself contributing to aging. Oxidative stress, which is a detrimental consequence of free radical accumulation in the body, is a significant factor in aging. Consequently, effective mitigation of free radicals in the human body can be achieved by ROS or through the formation of stable compounds via reactions with ROS, thereby potentially attenuating the aging process.

### 3.4 DEGs Involved in Longevity Pathway

**FIGURE 5:**
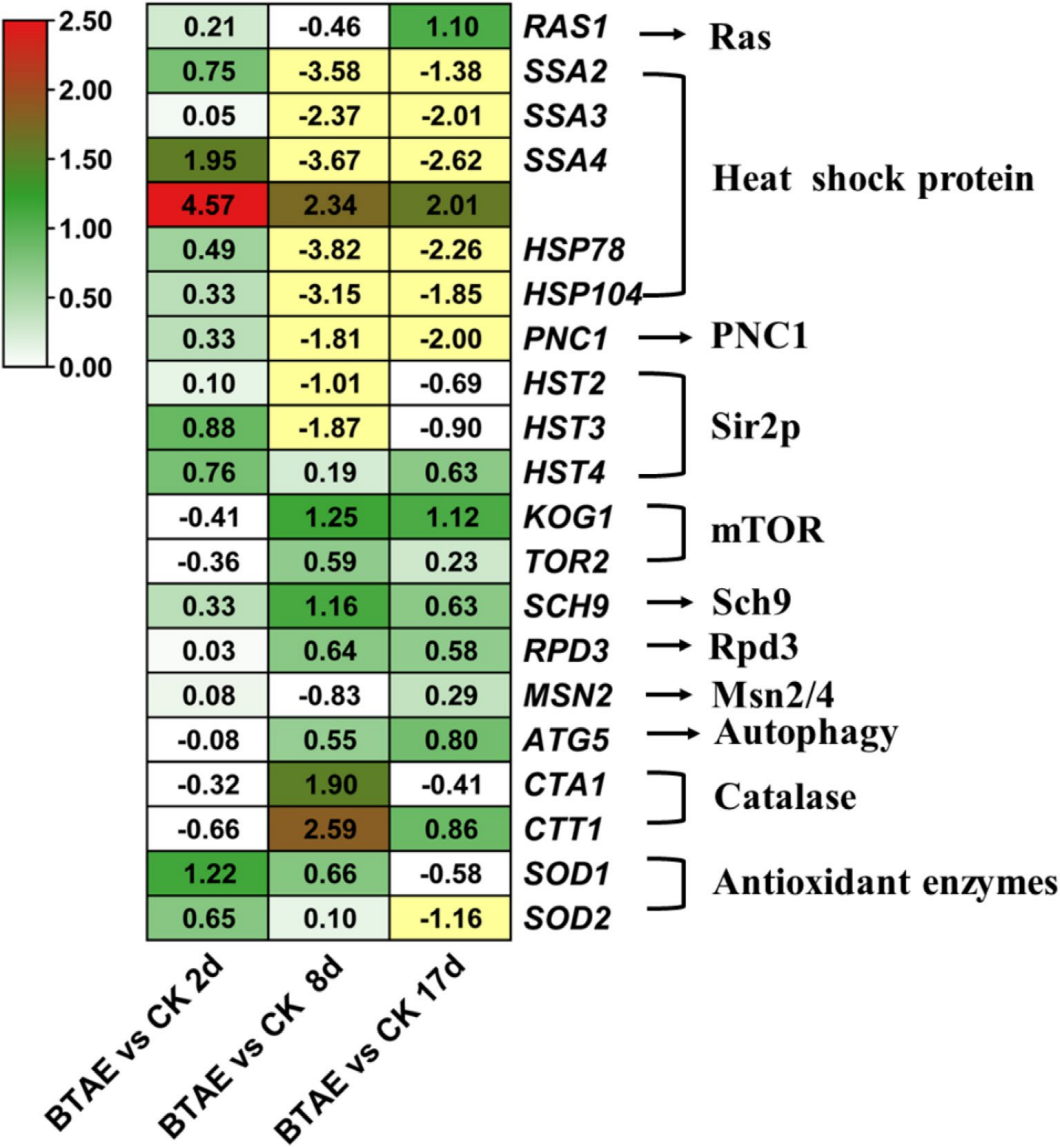
Longevity regulating pathway which differentially expressed genes were involved in.

**FIGURE 6:**
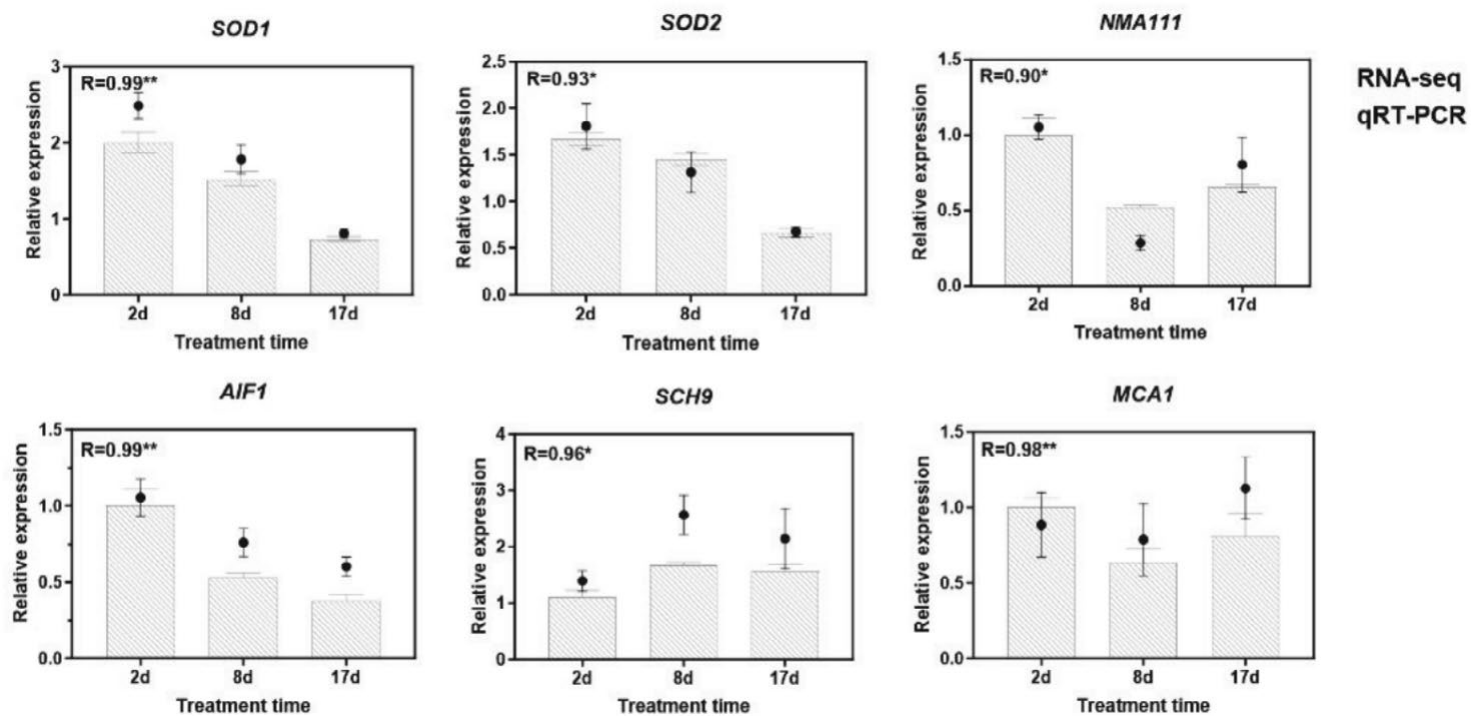
qRT‐PCR confirmation of six candidate genes. Data are presented as mean ± SD (*n* = 3 biological replicates per group).

Twenty‐one known or putative longevity‐regulating pathway‐related genes, including *rat sarcoma homolog* (*Ras*), heat shock protein (*stress‐seventy subfamily A2* (*SSA2*), *SSA3*, *SSA4*, *HSP78*, and *HSP104*), *pyrazinamidase and nicotinamidase 1* (*PNC1*), mTOR (*kontroller of growth 1* (*KOG1*) and *TOR2*), and *SCH9* (Table S6 and Figure 5). Most heat shock protein‐ and silent information regulator 2p (Sir2p)‐related genes were significantly down‐regulated in the BTAE vs. CK groups at 8 and 17 days, whereas genes related to mTOR, Sch9, reduced potassium dependency 3 (Rpd3), and autophagy were significantly up‐regulated. Genes encoding SOD were significantly up‐regulated in the BTAE vs. CK groups on days 2 and 8. In addition to affecting antioxidant gene expression, BTAE regulates the expression of aging‐ and apoptosis‐related genes. Our results showed that during the first 8 days of growth, BTAE increased the expression of antioxidant‐related genes (*SOD1*, *SOD2*, *catalase A1* [*CTA1*], and *catalase T1* [*CTT1*]). The up‐regulated *SOD1* and *SOD2* genes may be crucial for ROS scavenging, redox balance maintenance, and delayed aging in *S. cerevisiae* treated with BTAE. Similar results were observed in *S. cerevisiae* treated with *Polyalthia longifolia* leaf extract (Hemagirri and Sasidharan 2022). In order to validate the expression levels of the DEGs from transcriptome sequencing, the expression patterns of six DEGs, including antioxidant‐related genes (*SOD1* and *SOD2*) and aging‐related genes (*nuclear mediator of apoptosis 111* [*NMA111*], *apoptosis‐inducing factor 1* [*AIF1*], *SCH9*, and *meta‐caspase 1* [*MCA1*]), were analyzed using qRT‐PCR. The qRT‐PCR results showed a significant correlation with the transcriptomic sequencing data (*p* < 0.05), confirming the reliability of the transcriptomic sequencing results (Figure 6). Yeast programmed cell death is a gene‐controlled process of autonomous cell death that is crucial for maintaining the normal physiological functions of cells and organisms (Yan et al. 2023). Proteases have been shown to play a crucial role in this process as they can degrade specific proteins, thereby affecting the survival or death of cells. NMA111 and MCA1 are two specific proteases involved in regulating programmed cell death in yeast. Under stress conditions, the overexpression of *NMA111* promotes cell apoptosis, whereas cells lacking *NMA111* exhibit a stronger survival ability in the heat shock response than wild‐type cells (Zhu et al. 2016, 2017). AIF1 may interact with other apoptosis‐related proteins to regulate mitochondrial membrane permeability. Studies have shown that changes in the expression level of the *AIF1* gene can affect the sensitivity of yeast cells to apoptosis‐inducing factors. If the expression of the *AIF1* gene is inhibited, yeast cells may exhibit delayed or reduced apoptosis when faced with stimuli that trigger apoptosis (Chin et al. 2014). These genes are markers of programmed cell death and are involved in oxidative stress. The BTAE caused decreased *NMA111* and *AIF1* gene expression, suggesting the inhibitory effect of BTAE on apoptosis. Methuselah (MTH), a gene discovered in *Drosophila melanogaster* , plays an important role in physiological processes and lifespan regulation in *Drosophila*. In *Drosophila*, mutation of the MTH gene extends lifespan by 35% and increases tolerance to extreme environments (Lin et al. 1998). Interestingly, we also found an MTH gene (*MTH1*) in yeast that was significantly inhibited by BTAE, indicating that *MTH1* may also be a key gene regulating aging in yeast. The gene expression patterns of most lifespan regulatory pathways are opposite to those of lifespan; however, this may be a balancing mechanism in yeast that requires further research. BTAE enhanced *S. cerevisiae* 's antioxidant activity, which is demonstrated by its effects on yeast antioxidant enzyme activity, oxidative stress, and gene expression.

### 3.5 ROS Contents and Antioxidant Enzyme Activity in BTAE‐Treated *S. cerevisiae*

**FIGURE 7:**
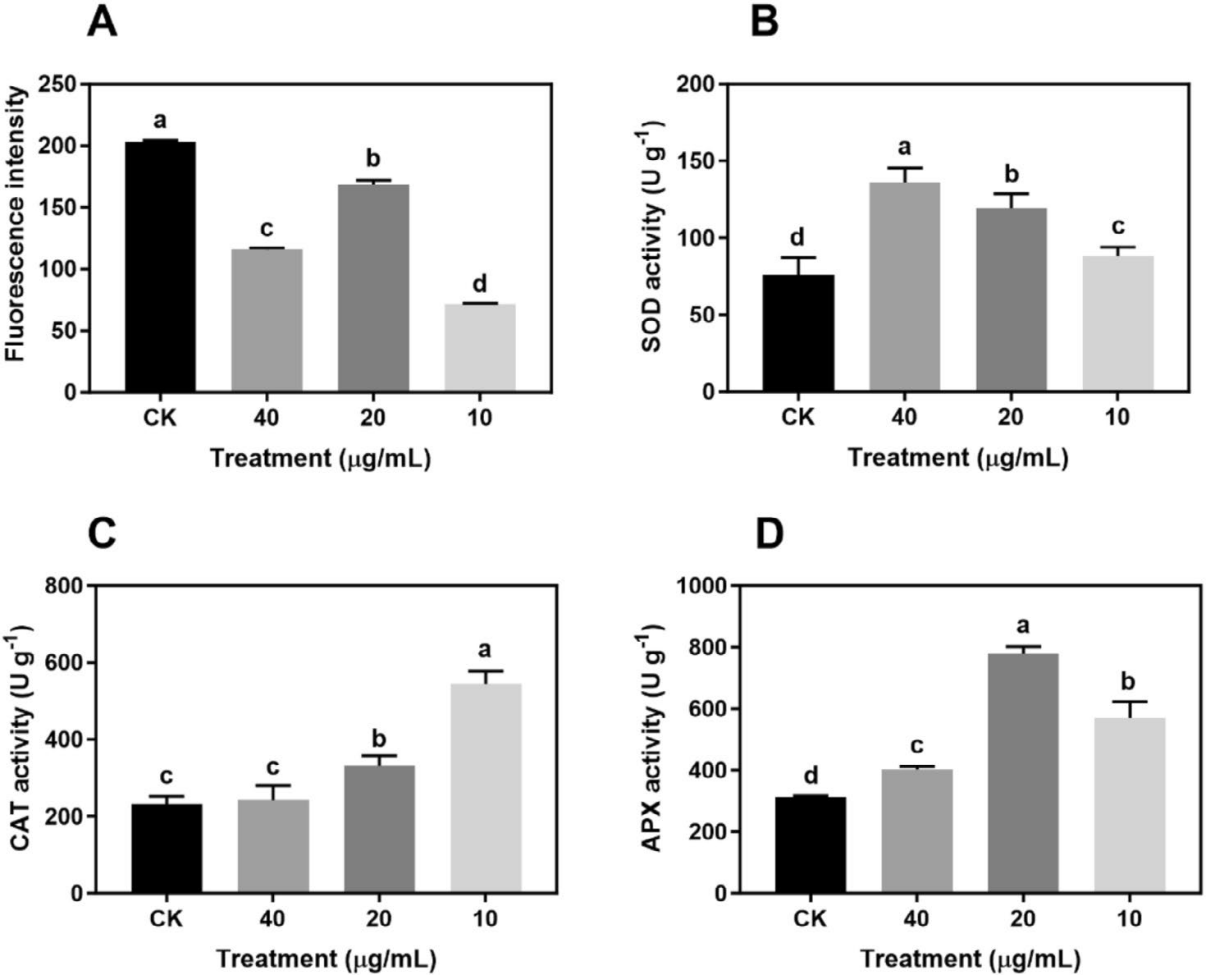
Effects of BTAE treatment on intracellular ROS levels (A) and the activities of SOD (B), CAT (C), and APX (D) in yeast (BY4742). Results were expressed as means ± SD, *n* = 3 experiments. Different letters represent significant differences at *p* < 0.05.

In order to assess whether the enhanced survival of *S. cerevisiae* following BTAE treatment was associated with decreased ROS generation, ROS levels in these cells were measured. The levels of ROS produced as by‐products in the biological systems were assessed using H_2_DCFDA. BTAE treatment notably decreased ROS levels, with 20 μg/mL being the most effective concentration (Figure 7A). Additionally, the activities of antioxidant enzymes, including SOD, CAT, and APX, were assessed. Notably, while SOD, CAT, and APX are conserved ROS detoxification enzymes, APX is primarily found in plant systems. As illustrated in Figure 7B, there was a dose‐dependent enhancement in superoxide dismutase (SOD) activity in wild‐type *S. cerevisiae* treated with BTAE. When compared with the control group, the CAT activity in the BTAE groups significantly increased by 2.4‐fold (10 μg/mL) and 1.4‐fold (20 μg/mL), respectively (Figure 7C). However, the 40 μg/mL BTAE did not induce the activity of CAT compared to the control. As the concentration of BTAE increased, the APX activity of the three treatments significantly increased by 82.1%, 149.6%, and 28.6%, respectively, compared with that of control (Figure 7D). When cells are stimulated by external factors, excessive ROS are produced inside the cells, leading to oxidative stress. If the external stimuli are too strong, the endogenous antioxidant system of cells may become overwhelmed, resulting in the inability of ROS clearance to maintain the rate of ROS production. Excessive accumulation of ROS can cause serious damage to cells, thus disrupting their structure and function, including damaging the integrity of cell membranes, causing protein denaturation and DNA strand breaks, and ultimately leading to apoptosis or necrosis (Ma et al. 2009). Recent studies have elucidated mechanistic links between flavonoids and ROS in cellular processes. Flavonoids possess phenolic hydroxyl groups that can directly scavenge ROS through hydrogen atom transfer (HAT) or single electron transfer (SET) mechanisms (Zheng et al. 2022). The catechins identified in the BTAE (e.g., (−)‐epigallocatechin‐3‐gallate (EGCG)) contain ortho‐dihydroxy groups that chelate transition metals, thereby inhibiting Fenton's reaction‐mediated ROS generation (Bernatoniene and Kopustinskiene 2018). Specifically, EGCG downregulates NADPH oxidase activity while upregulating endogenous antioxidant enzymes such as SOD and CAT through Nrf2/ARE pathway activation (Mokra et al. 2022). Notably, theaflavins and theabrownins, key components of BTAE, have been shown to modulate oxidative stress and gut microbiota in aging models, which is consistent with our findings on redox homeostasis and lifespan extension (Cai et al. 2021). This dual action modulates redox homeostasis, which is critical for cell viability and CLS extension. The enhanced antioxidant effects align with the free radical theory of aging proposed by Harman, in which reduced ROS levels correlate with lifespan extension across species. This is supported by the observations that long‐lived mutants universally exhibited decreased ROS production and upregulated antioxidant enzymes. Notably, BTAE‐induced SOD/CAT upregulation mirrors genetic interventions (e.g., SOD overexpression or Sch9 pathway inactivation) that extend yeast lifespan through similar redox homeostasis mechanisms. Furthermore, the observed enzymatic activation parallels the protective effects of specific flavonoids (e.g., quercetin and kaempferol) against oxidative damage, which operate through both direct ROS scavenging and indirect regulation of stress response pathways. These dual mechanisms collectively reduce macromolecular damage (DNA, proteins, and lipids), which is a key factor in regard to maintaining cell viability and extending CLS. SOD is the first line of defense against oxidative stress in cells and can effectively eliminate superoxide anions, maintain low levels of ROS, protect cells from oxidative damage, and delay aging‐related cellular functional decline (Hemagirri and Sasidharan 2022). In addition, SOD1 and SOD2 are closely related to yeast lifespan regulation, and knocking out these two genes leads to rapid yeast death (Ma et al. 2009). Theabrownin improved learning, memory, and liver oxidative stress (SOD, GSH‐Px, and MDA) and increased the relative abundance of *Lactobacillus_murinus* and *Bacteroides_acidifacien*s in aging mice, thereby preventing and delaying aging (Lei et al. 2022). Thearubigin polymers (TRs) have demonstrated significant antioxidative properties that counteract aging by scavenging reactive oxygen species (ROS) like O₂^−^, ·OH, and H₂O₂, which accumulate during metabolism and cause oxidative damage; in vitro studies confirm TRs protect against ROS‐induced DNA breaks (Li et al. 2013; Peng et al. 2009). Transcriptomic data and antioxidant enzyme assays demonstrated synergistic mechanistic convergence. BTAE simultaneously upregulated SOD/CAT/APX‐coding genes (e.g., *SOD1*, *SOD2*, *CAT1*) while enhancing their enzymatic activities, thereby creating a positive feedback loop for ROS clearance. This dual regulation at both transcriptional and functional levels explains how BTAE maintains redox homeostasis during yeast aging, as evidenced by reduced oxidative stress markers (Figure 7A) and an extended chronological lifespan (CLS). In this study, BTAE reduced oxidative stress in *S. cerevisiae* by enhancing antioxidant enzymes (SOD, CAT, and APX), suggesting that it delays yeast aging via its antioxidant effects.

## 4 Conclusion

The findings of this study have demonstrated that BTAE has significant anti‐aging effects on *Saccharomyces cerevisiae* (yeast). BTAE extends the chronological lifespan (CLS) of yeast by mitigating oxidative stress and maintaining a balanced oxidation‐antioxidant system, thereby protecting mitochondrial function and cellular integrity. This anti‐aging activity is achieved through two mechanisms: up‐regulation of key endogenous antioxidant genes (e.g., *SOD1*, *SOD2*, *CTA1*, and *CTT1*), which enhance cellular defense against reactive oxygen species (ROS), and down‐regulation of apoptosis‐related genes (e.g., *NMA111*, *AIF1*, and *MTH1*), which reduce programmed cell death and promote cellular survival. Additionally, BTAE modulated critical pathways involved in the regulation of aging, including oxidative phosphorylation, autophagy, and the biosynthesis of secondary metabolites, further supporting its role in delaying cellular aging. The polyphenol‐rich composition of BTAE, particularly its high theabrownin, thearubigin, and catechin contents, is likely responsible for its potent antioxidant and anti‐aging properties. These findings there highlight the potential of natural polyphenol‐rich extracts such as BTAE as valuable sources of anti‐aging compounds. This study provides insights into the molecular mechanisms underlying the anti‐aging effects of BTAE and lays the groundwork for further research into its application in anti‐aging therapies and the development of natural anti‐aging agents to delay aging and extend longevity in higher organisms, including humans.

## Author Contributions

**Jie Li:** conceptualization (equal), data curation (equal), funding acquisition (equal), investigation (equal), writing – original draft (equal). **Qiyang Chen:** investigation (equal), writing – review and editing (equal). **Fuliang Xiao:** data curation (equal). **Juan Yang:** data curation (equal), formal analysis (equal). **Sijia Zhou:** validation (equal). **Min Tang:** resources (equal). **Yujia Hou:** resources (equal). **Xiuming Zhai:** conceptualization (equal), funding acquisition (equal), writing – review and editing (equal).

## Conflicts of Interest

The authors declare no conflicts of interest.

## Supporting information


Data S1.

## Data Availability

Data will be made available on request.
